# Supplementary material for: Circulating Tumor DNA Predicts Early Recurrence Following Locoregional Therapy for Oligometastatic Colorectal Cancer
Source: Cancers (Basel). 2024 Jun 29;16(13):2407. doi: 10.3390/cancers16132407 (PMC11240520; doi:10.3390/cancers16132407)
Supplement: Supplementary file 1 [file cancers-16-02407-s001.zip › cancers-3084322-supplementary.pdf]

Supplementary Material

Circulating Tumor DNA Predicts Early Recurrence Following Locoregional Therapy for Oligometastatic Colorectal Cancer

Conor D.J. O'Donnell, Nikolas Naleid, Teerada Siripoon, Kevin G. Zablonski, Michael H. Storandt, Jennifer E. Selfridge, Chris L. Hallemeier, Madison L. Conces, Krishan R. Jethwa, David L. Bajor, Cornelius A. Thiels, Susanne G. Warner and Patrick P. Starlinger, Thomas D. Atwell, Amit Mahipal, Zhaohui Jin*

*** Correspondence:** jin.zhaohui@mayo.edu

Table S1: Univariate (A) and multivariate (B) analysis to assess prognostic factors for Disease-Free Survival in Entire Cohort

A)

Variable	HR	95%CI	P value
Sex<br>- Female<br>- Male	 Reference<br>1.97	 <br>1.13-3.42	 <br>**0.016**
Age<br>- < 65<br>- ≥ 65	 Reference<br>0.68	 <br>0.37-1.23	 <br>0.198
Tumor sidedness<br>- Left<br>- Right	 Reference<br>0.72	 <br>0.37-1.39	 <br>0.322
Pattern of metastatic disease<br>- Synchronous metastases<br>- Metachronous metastases			

	Reference<br>0.76	0.44-1.31	0.326
Pre-intervention ctDNA<br>- Negative<br>- Positive	Reference<br>1.91	0.53-6.83	0.320
Location of isolated metastases<br>- Liver<br>- Lung<br>- Other	Reference<br>1.79<br>0.48	0.76-4.22<br>0.20-1.12	0.185<br>0.090
Location of isolated metastases<br>- Liver<br>- Other	Reference<br>0.76	0.40-1.44	0.394
Post-intervention ctDNA<br>- Negative<br>- Positive	Reference<br>2.69	1.59-4.55	**<0.001**
Neoadjuvant chemotherapy<br>- < 6 months<br>- ≥ 6 months	Reference<br>2.21	1.13-4.31	**0.020**
Local treatment<br>- Resection<br>- Other	Reference<br>0.95	0.55-1.64	0.860
Post-intervention chemotherapy<br>- No			

- Yes	Reference<br>0.99	0.59-1.68	0.975
Post-intervention CEA<br>- Negative<br>- Positive	Reference<br>1.56	0.84-2.89	0.158
KRAS/BRAF/NRAS mutation<br>- Negative<br>- Positive	Reference<br>1.07	0.62-1.85	0.796

B)

Variable	HR	95%CI	P value
Sex<br>- Female<br>- Male	Reference<br>2.04	1.16-3.59	**0.013**
Post-intervention ctDNA<br>- Negative<br>- Positive	Reference<br>2.68	1.54-4.64	**<0.001**
Neoadjuvant chemotherapy<br>- < 6 months<br>- ≥ 6 months	Reference<br>1.33	0.66-2.68	0.430

Figure S1: Disease-Free Survival from time of post-intervention ctDNA sampling

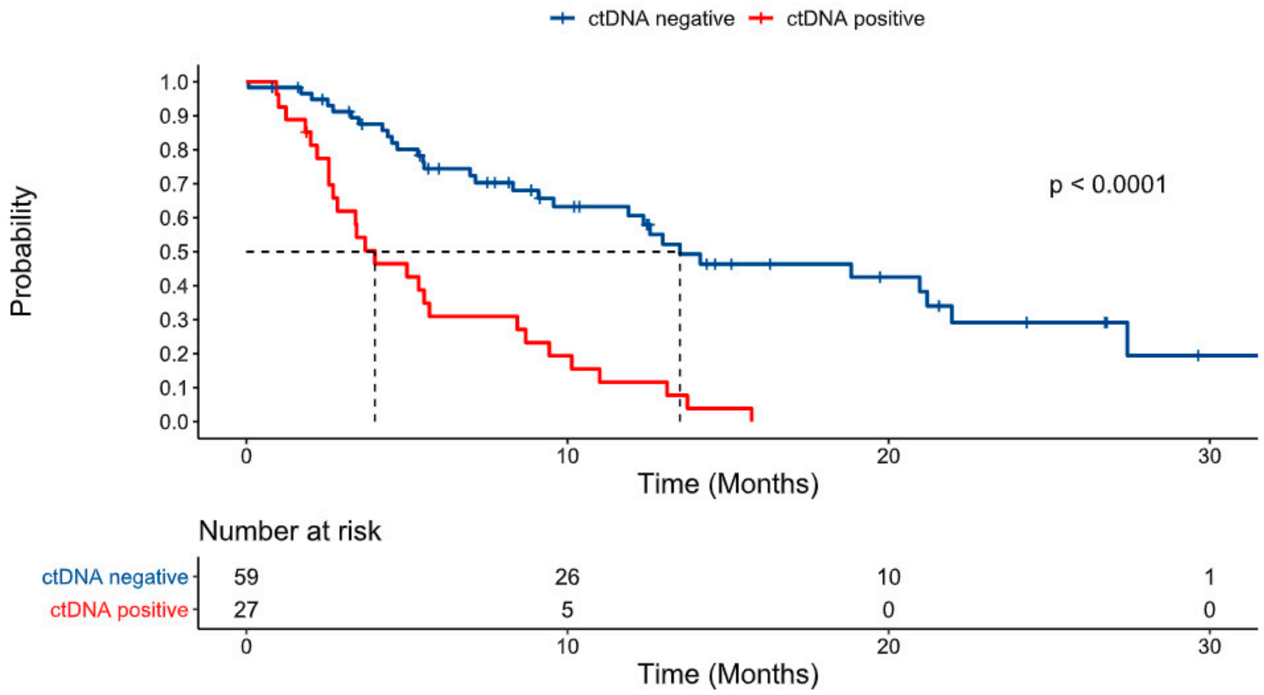

	Event	Median DFS (month)	95%CI	P value
ctDNA negative (N=59)	31 (52.5%)	13.50	11.90-NA	**<0.0001**
ctDNA positive (N=28)	27 (96.4%)	4.00	2.70-8.70	

Table S2: Location of recurrence after locoregional intervention for oligometastatic colorectal cancer according to ctDNA status

Post-Intervention ctDNA	Site of Recurrence				
	Liver	Lung	Abdominal node/peritoneal	Multiple sites	Total
ctDNA Negative	14 (45%)	6 (19%)	3 (10%)	8 (26%)	31
ctDNA positive	11 (43%)	7 (27%)	3 (12%)	5 (19%)	26

P=0.874 for difference in proportions

Table S3: Univariate (A) and Multivariate (B) analysis to assess prognostic factors for Disease-Free Survival measured from time of ctDNA test

A)

Variable	HR	95%CI	P value
Sex<br>- Female<br>- Male	Reference<br>1.58	0.92-2.70	0.094
Age<br>- < 65<br>- $\geq$ 65	Reference<br>0.56	0.31-1.03	0.061
Tumor sidedness<br>- Left<br>- Right	Reference<br>0.57	0.29-1.10	0.094

Pattern of metastatic disease<br>- Synchronous metastases<br>- Metachronous metastases	Reference<br>0.47	0.26-0.84	**0.011**
Pre-intervention ctDNA<br>- Negative<br>- Positive	Reference<br>1.83	0.51-6.56	0.352
Location of isolated metastases<br>- Liver<br>- Lung<br>- Other	Reference<br>1.55<br>0.55	0.65-3.65<br>0.23-1.30	0.321<br>0.171
Location of isolated metastases<br>- Liver<br>- Other	Reference<br>0.81	0.43-1.55	0.532
Post-intervention ctDNA<br>- Negative<br>- Positive	Reference<br>4.14	2.36-7.26	**<0.001**
Neoadjuvant chemotherapy<br>- < 6 months<br>- ≥ 6 months	Reference<br>2.01	1.03-3.91	**0.040**
Local treatment<br>- Resection			

- Other	Reference<br>1.28	0.74-2.22	0.377
Post-intervention chemotherapy<br>- No<br>- Yes	Reference<br>1.12	0.66-1.91	0.680
Post-intervention CEA<br>- Negative<br>- Positive	Reference<br>1.61	0.87-3.00	0.130
KRAS/BRAF/NRAS mutation<br>- Negative<br>- Positive	Reference<br>1.06	0.61-1.83	0.837

B)

Variable	HR	95%CI	P value
Pattern of metastatic disease<br>- Synchronous metastases<br>- Metachronous metastases	Reference<br>0.46	0.25-0.83	**0.010**
Post-intervention ctDNA<br>- Negative<br>- Positive	Reference<br>3.87	2.13-7.01	**<0.001**

Neoadjuvant chemotherapy<br>- < 6 months<br>- ≥ 6 months	Reference<br>1.34	0.66-2.74	0.414

Figure S2: Forest Plot of Multivariable Analysis of Prognostic Factors for Disease-Free Survival from ctDNA Assessment

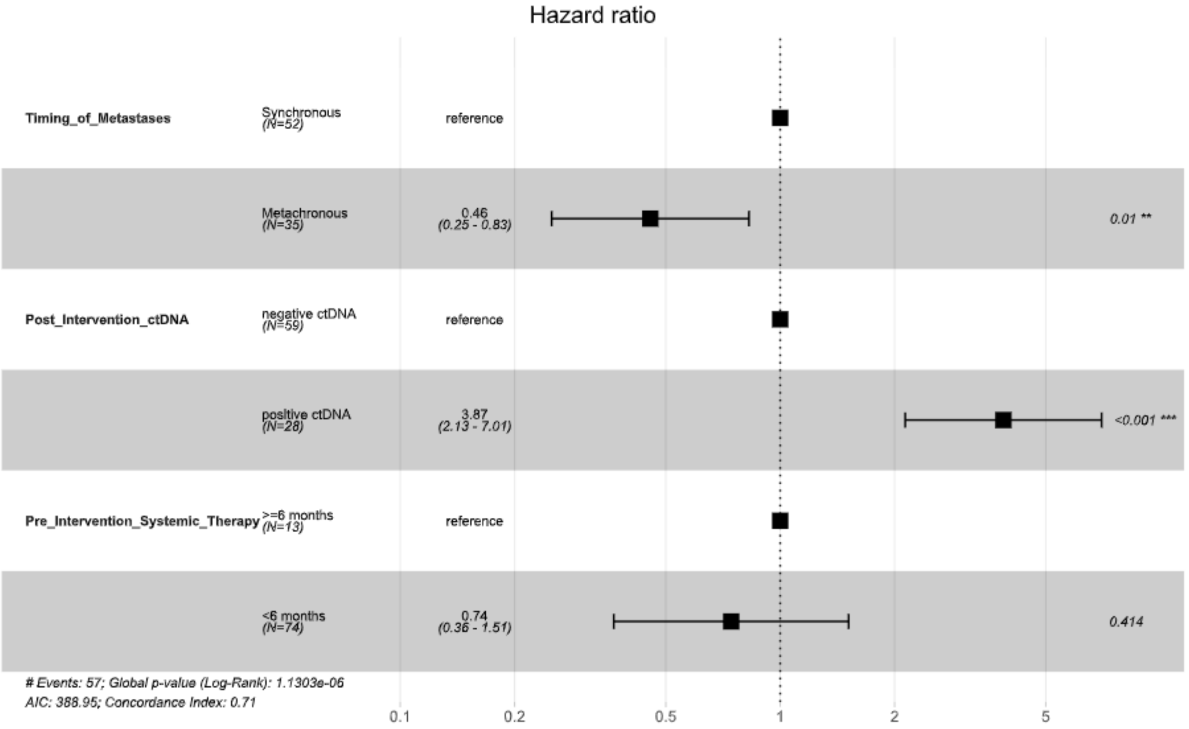

Figure S4: Overall Survival by Post-Intervention ctDNA Status

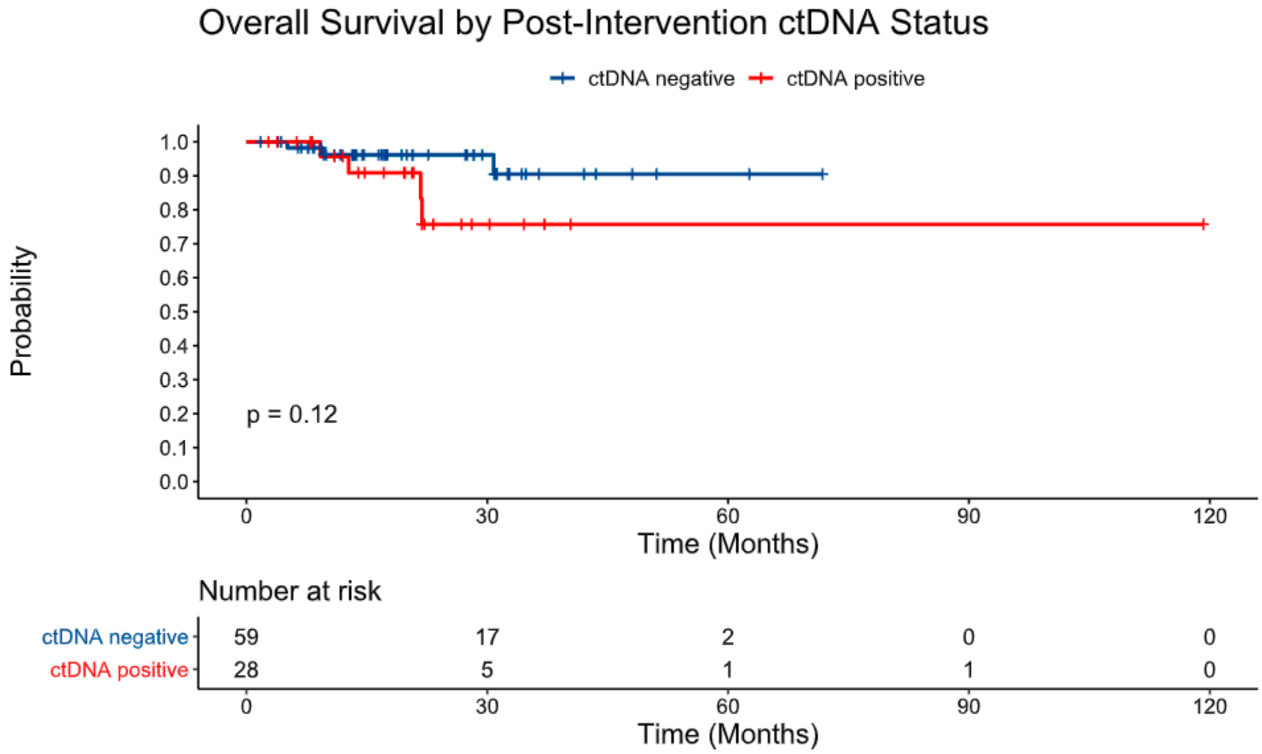

	Event	Median DFS (month)	95%CI	P value
ctDNA Negative (N=59)	3 (5.1%)	NA	NA-NA	0.122
ctDNA Positive (N=28)	4 (14.2%)	NA	NA-NA	

Table S4: Characteristics of patients with and without post-intervention systemic therapy

Characteristics	Without post-intervention systemic therapy (N=38)	With post-intervention systemic therapy (N=49)	P value
Sex			
- Female	20 (52.6%)	19 (38.8%)	0.197
- Male	18 (47.4%)	30 (61.2%)	
Age			
- < 65	25 (65.8%)	37 (75.5%)	0.320
- ≥ 65	13 (34.2%)	12 (24.5%)	
Tumor sidedness			
- Left	26 (68.4%)	38 (77.6%)	0.338
- Right	12 (31.6%)	11 (22.4%)	
Pattern of metastatic disease			
- Synchronous metastases	21 (55.3%)	31 (63.3%)	0.450
- Metachronous metastases	17 (44.7%)	18 (36.7%)	
Pre-intervention ctDNA			
- Negative	2 (16.7%)	7 (33.3%)	0.301
- Positive	10 (83.3%)	14 (66.7%)	
- Unknown			

	26	28	
Location of isolated metastases<br>- Liver<br>- Lung<br>- Other	27 (71.1%)<br>5 (13.2%)<br>6 (15.8%)	41 (83.7%)<br>3 (6.1%)<br>5 (10.2%)	0.347
Location of isolated metastases<br>- Liver<br>- Other	27 (71.1%)<br>11 (28.9%)	41 (83.7%)<br>8 (16.3%)	0.158
Neoadjuvant chemotherapy<br>- < 6 months<br>- ≥ 6 months	31 (81.6%)<br>7 (18.4%)	43 (87.8%)<br>6 (12.2%)	0.423
Local treatment<br>- Resection only<br>- Other	25 (65.8%)<br>13 (34.2%)	28 (57.2%)<br>21 (42.9%)	0.412
Post-intervention ctDNA<br>- Negative<br>- Positive	26 (68.4%)<br>12 (31.6%)	33 (67.3%)<br>16 (32.7%)	0.915
Post-intervention CEA<br>- Negative<br>- Positive<br>- Unknown	18 (64.3%)<br>10 (35.7%)<br>10	28 (71.8%)<br>11 (28.2%)<br>10	0.513

Table S5: Univariate analysis to assess prognostic factors for DFS in 8-week post-intervention ctDNA negative patients

Variable	HR	95%CI	P value
Sex<br>- Female<br>- Male	Reference<br>1.78	0.67-4.72	0.245
Age<br>- < 65<br>- ≥ 65	Reference<br>0.69	0.26-1.87	0.472
Tumor sidedness<br>- Left<br>- Right	Reference<br>0.91	0.33-2.47	0.852
Pattern of metastatic disease<br>- Synchronous metastases<br>- Metachronous metastases	Reference<br>0.38	0.14-1.05	**0.061**
Pre-intervention ctDNA<br>- Negative<br>- Positive	Reference<br>0.33	0.06-2.54	0.330
Location of isolated metastases<br>- Liver<br>- Other	Reference<br>1.45	0.52-4.05	0.481

Neoadjuvant chemotherapy<br>- < 6 months<br>- ≥ 6 months	Reference<br>1.93	0.24-15.29	0.535
Local treatment<br>- Resection<br>- Other	Reference<br>1.57	0.54-4.56	0.404
Post-intervention chemotherapy<br>- No<br>- Yes	Reference<br>0.60	0.22-1.68	0.335
New class adjuvant chemotherapy<br>- No<br>- Yes	Reference<br>1.17	0.12-11.51	0.888
Post-intervention CEA<br>- Negative<br>- Positive	Reference<br>2.20	0.57-8.58	0.255

Figure S5: Disease-Free Survival by Post-Intervention Chemotherapy Status amongst ctDNA negative patients

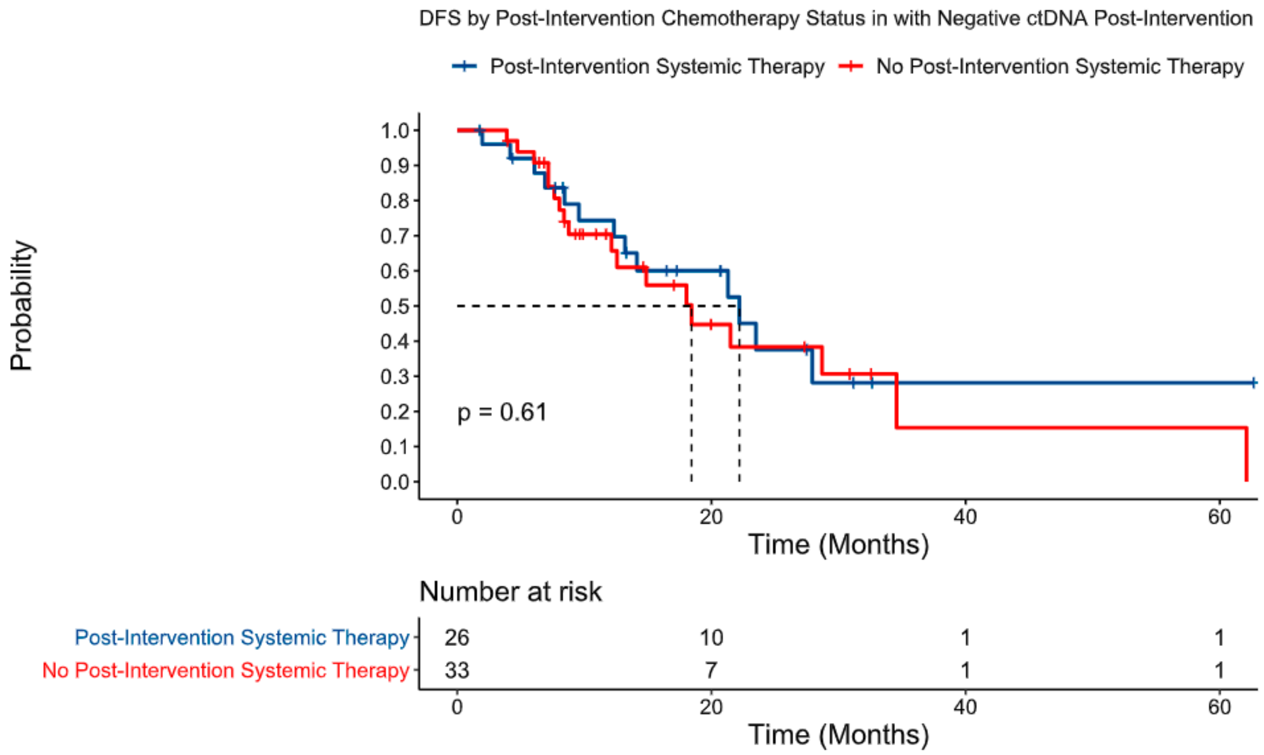

	Event	Median DFS (month)	95%CI	P value
No Post-Intervention Systemic Therapy (N=26)	13 (50%)	22.20	13.20-NA	0.613
No Post-Intervention Systemic Therapy (N=33)	18 (54.5%)	18.43	12.57-NA	
